# Knowledge and practice of spirometry among pediatricians in Riyadh, Kingdom of Saudi Arabia

**DOI:** 10.4103/1817-1737.39637

**Published:** 2008

**Authors:** Muslim Mohammed Al-Saadi

**Affiliations:** *Department of Pediatrics, College of Medicine, King Khalid University Hospital, King Saud University, Riyadh, Saudi Arabia*

**Keywords:** Knowledge, pediatricians, practice, spirometry

## Abstract

**BACKGROUND:**

Spirometry is the most basic, widely used and effort-dependent pulmonary function test. It assesses the lung volumes and flows, and is ideally suited to describe the effects of restriction or obstruction on lung function. Therefore, keeping in view the clinical applications of spirometry, this study attempts to explore the knowledge and practice about spirometry among pediatricians.

**MATERIALS AND METHODS:**

A questionnaire-based study was conducted across multiple centers in various hospitals in Riyadh, Saudi Arabia. The structured questionnaire, based upon knowledge and practice of spirometry, was distributed to 150 pediatricians in the various tertiary care hospitals in the metropolitan area of Riyadh.

**RESULTS:**

Ninety-four percent of 113 pediatricians agreed that spirometry is a valuable tool in pediatric clinical practice. However, knowledge relating to spirometry was lacking among pediatricians, and about 86% of the study population did not demonstrate up-to-date knowledge of spirometry in pediatrics. Only 11% of pediatricians were very confident in interpreting spirometry results. No statistically significant association was observed between the distribution of responses relating to knowledge and practice of spirometry and the study variables including academic position, duration of practicing experience and number of patients attended daily.

**CONCLUSION:**

The results indicated that pediatricians in Riyadh were lacking adequate knowledge about the clinical applications of spirometry in their daily clinical practice. Hence, it was suggested that pediatricians should attend periodical training, workshops and continuous medical education programmes to enhance their knowledge. This should especially be performed during their pediatric residency training programmes, as spirometry is one of the essential components in clinical practice.

In the past few decades, there has been an increase in the incidence of respiratory illnesses and asthma in children globally.[1] This could be due to several factors, including environmental pollution, passive smoking and modernization. Timely and suitable diagnosis is important for the proper management of respiratory ailments. Among the various investigation modalities available, pulmonary function tests (PFT) are a valuable tool for the assessment of lung function and are being increasingly used for clinical assessment and monitoring childhood respiratory diseases in preschool children. However, objective assessment of pulmonary function in children represents a major challenge.[1]

Spirometry is the most basic, extensively used, effort-dependent PFT. Spirometric measurements characteristically assess lung volumes and flows and are ideally suited to describe the effects of restriction or obstruction on lung function[2] in adults and older children in bronchial asthma and other respiratory conditions.[3] The assessment of pulmonary function with a spirometer has become common practice in patient settings. It is now regarded as a vital component of any respiratory medical surveillance programme. Spirometry is used to determine the extent of impairment and assess the response to treatment. PFT has assumed a key role in epidemiological studies: investigating the incidence, natural history and causality of environmental lung disease.[4] Periodic re-testing of personnel can detect pulmonary disease in its earliest stages, where corrective measures are more likely to be beneficial. Such interventions could include improvement in better clinical setting. Spirometry is often performed to assess the risk of anesthetic procedure.[2] Moreover, it is important in confirming the diagnosis and assessing the severity of the disease.[5] Hence, PFT for lungs is comparable to that of electrocardiography (ECG) for heart.[6] Furthermore, it has been well acknowledged that spirometry can be achieved in children younger than 6 years of age.[7–9] In addition, the increasing use of spirometry in young children will require some revision of quality control currently described in adults and older children[1] and ATS/ERS guidelines for spirometry quality control in young children.[3] Spirometry is often regarded as a simple, noninvasive screening test, but careful consideration should be given to a number of aspects, including optimal test performance, adherence to standard acceptability and repeatability criteria, and accurate and proper interpretation of the results. Routine use of spirometry by general practitioners (GPs) is now feasible with widespread availability of computerized portable spirometers.[10] In the face of this evidence, factors like lack of training, knowledge and access to a well-maintained spirometer are causing underutilization of spirometry.[11] To the best of our knowledge, this is the first study to address the knowledge and practice on spirometry among pediatricians in the Kingdom of Saudi Arabia and worldwide. Therefore, this study was carried out with the objective to assess the knowledge and practice of spirometry by pediatricians in their clinical practice at the referral hospitals of Riyadh city of Kingdom of Saudi Arabia.

## Materials and Methods

### Study design

The present study was carried out during the period January-November 2007. The study subjects were 150 pediatricians from various tertiary care hospitals in the metropolitan area of Riyadh, Kingdom of Saudi Arabia. These pediatricians, who were postgraduate fellows and faculty members, were selected randomly based on their informed consent.

### Survey instrument

A pre-tested, structured questionnaire was designed and administered to collect information about the knowledge and practice of spirometry among pediatricians. The first series of questions concerned personal characteristics such as age, sex, qualification, position, experience (number of years practicing) and total number of patients visiting the clinics. These variables were used to stratify the study subjects, as these factors might influence the use of spirometry. The second series of questions focused on knowledge and practices towards the use of spirometry, which were related to the outcomes of this study. These questions were geared to assess the pediatric practitioner's knowledge about the use of spirometry and its clinical application in the management of respiratory and other related medical illnesses. The questions were mostly clinical indications for the use, application and practice of spirometry. In particular, these questions focussed on whether or not pediatricians would suggest or arrange spirometry for pediatric patients and also attempted to identify factors that enhance and limit the use of spirometry by pediatric practitioners. The responses were measured on 4-point, 3-point and binary scales.

### Data management and statistical analyses

Data were entered in MS-Excel spreadsheets and analyzed using SPSS Statistical Software (version 11.0). Descriptive statistics (mean, standard deviation and percentage) were used to summarize the data. Chi-squared test was used (i) to assess the association between two categorical variables and also (ii) to observe the statistically significant distribution of responses (observed from the data) by comparing with expected values of responses, which were hypothesized to be distributed uniformly.

## Results

Out of the 150 questionnaires distributed, filled-in questionnaires were received from 113 pediatricians with a response rate of 75.3%. The socio-demographic characteristics and other study variables of these subjects are given in Table 1.

**Table 1:** Socio-demographic characteristics of study sample

Variables	Mean ± SD
Age	35.6 ± 3.4
Practicing experience	9.1 ± 6.7
Approximate number of patients visiting daily	10.1 ± 9.3
Approximate number of visiting children per day who suffer from acute respiratory disease	5.6 ± 4.7
Approximate number of visiting children per day who suffer from chronic respiratory disease	3.8 ± 3.3
	**No (%)**
Gender (male) (n = 113)	64 (55.7)
Current position (n = 111)	
Consultant	44 (38.3)
Registrar	27 (23.5)
Resident	40 (34.8)

### Knowledge

The responses to 10 statements on knowledge of spirometry are shown in Table 2. The proportions of 3-point scale responses to 8 statements indicated that 37.6% to 56.6% of study subjects have stated spirometry as “very helpful”, “very important” and “very useful”. Conversely, 38% to 52.5% of pediatricians responded “fairly helpful”, “fairly important” and “fairly useful”, whereas 2.8% to 9.9% of them responded “not helpful”, “not important” and “not useful”. Although these 8 knowledge statements were simple, along with the mutually exclusive 3-point response scale, the above proportions indicate that all pediatricians were not having similar knowledge and complete knowledge relating to spirometry. A distribution of responses on 4-point scale [never (32.4%), rarely (19.6), sometimes (23.5%) and all the time (24.5%)] could be seen among these pediatricians for the statement, “While reviewing PFT how often you are provided with tracings of flow volume loop/flow time curve”. This also indicated that the level of knowledge was incomplete and varied widely. On the other hand, 86.1% of our study subjects have responded negatively to the statement, “Are you up-to-date with the current knowledge of spirometry in pediatrics”, which correlates with the distribution of responses to the other 9 statements of knowledge on PFT. The distributions of responses for all these 10 statements did not have uniformly distributed and statistically significantly different (*P* < 0.0001 for statements 1 to 8 and 10, and *P* = 0.04 for 9th statement).

**Table 2:** Distribution of responses towards the knowledge of pulmonary function tests

Questions related to knowledge of PFT		No. (%)
1.	How important you think is PFT in grading of respiratory disease? (n = 113)*	
	Not important	10 (8.9)
	Fairly important	43 (38)
	Very important	60 (53.1)
2.	How important you think is PFT in monitoring the course of a respiratory disease or condition of a patient? (n = 109)*	
	Not important	(6.4)
	Fairly important	45 (41.3)
	Very important	57 (52.3)
3.	How influential you think is the factor of patient compliance in PFT investigation especially in pediatrics? (n = 106)*	
	Not important	3 (2.8)
	Fairly important	43 (40.6)
	Very important	60 (56.6)
4.	How useful is PFT for diagnosis purpose in pediatrics? (n = 101)*	
	Not useful	10 (9.9)
	Fairly useful	53 (52.5)
	Very useful	38 (37.6)
5.	How useful is PFT in grading lung disease in pediatrics? (n = 102)*	
	Not useful	4 (3.9)
	Fairly useful	40 (39.2)
	Very useful	58 (56.9)
6.	How useful is PFT for prognosis purpose in pediatrics? (n = 100)*	
	Not useful	9 (9)
	Fairly useful	38 (38)
	Very useful	53 (53)
7.	How useful is PFT for monitoring lung diseases in pediatrics? (n = 100)*	
	Not useful	5 (5)
	Fairly useful	40 (40)
	Very useful	55 (55)
8.	How do you think PFT can influence your diagnosis based on clinical findings? (n = 113)*	
	Not helpful	9 (8.0)
	Fairly helpful	48 (42.5)
	Very helpful	56 (49.5)
9.	While reviewing PFT how often you are provided with tracings of flow volume loop/flow time curve? (n = 102)†	
	Never	33 (32.4)
	Rarely	20 (19.6)
	Sometimes	24 (23.5)
	All the time	25 (24.5)
10.	Are you up-to-date with the current medical knowledge of spirometry in pediatrics? (n = 108)*	
	No	98 (86.1)
	Yes	15 (13.9)

* *P* < 0.0001

† *P* = 0.04 (the responses are not uniformly distributed); PFT - Pulmonary function tests

### Practice

The distribution of responses on a 3-point scale to the 4 statements and a binary response to 3 statements towards the practice of spirometry is shown in Table 3. The proportion of responses to 3 statements of practice (3-point scale) reveals high heterogeneity where response to the first option (rare and once every three months) varies from 10% to 53%, second option (occasional and annually) varies from 17.5% to 45%, and the third option (whenever it is indicated, during follow-up visit) varies from 28.1% to 45%.

**Table 3:** Distribution of responses towards the practice of spirometry

Questions related to practice of spirometry		No. (%)
1.	How often do you use PFT in pediatric patients? (n = 114)*	
	Rare	62 (53.4)
	Occasional	20 (17.5)
	Whenever it is indicated	32 (28.1)
2.	How frequently are you suggesting spirometry for children suffering from acute respiratory disease? (n = 113)†	
	Not necessary	63 (55.7)
	As soon as patient is able	50 (44.3)
3.	How frequently are you suggesting spirometry for children suffering from chronic respiratory disease? (n = 109)*	
	Once every three months	11 (10)
	Annually	49 (45)
	During follow-up visit	49 (45)
4.	How frequently are you suggesting spirometry for children suffering from asthma? (n = 108)*	
	Once every three months	18 (16.7)
	Annually	46 (42.6)
	During follow-up visit	44 (40.7)
5.	How frequently are you suggesting spirometry for children on the follow-up treatment of chronic asthma? (n = 107)†	
	Annually	58 (54.2)
	During every follow-up visit	49 (45.8)
6.	Which one of the following test you would like to conduct in asthmatic children? (n = 112)†	
	Complete spirometry	59 (52.70
	Peak expiratory flow only	53 (47.3)
7.	How confident are you in interpreting PFT reports of pediatric patients? (n = 108)*	
	Not confident	54 (50)
	Fairly confident	42 (38.9)
	Very confident	12 (11.1)

* *P* < 0.001 (the responses are not uniformly distributed)

† *P* > 0.05 (the responses are uniformly distributed)

The proportion of responses to the statement, “How confident are you in interpreting PFT reports of pediatric patients”, was 50% (not confident), 38.9% (fairly confident) and 11.1% (very confident), which shows that pediatricians make a restricted use of PFT in their clinical practice. Moreover, the responses “not necessary” (55.7%) and “as soon as patient is able” (44.3%) were observed towards the question, “How frequently do you suggest spirometry for children suffering from acute respiratory disease”. Similar responses “annually” (54.2%) and “during every follow-up visit” (45.8%) were observed for the question, “How frequently do you suggest spirometry for the children on the follow-up treatment of chronic asthma”. The responses to these two questions about the practice of spirometry in treating both acute and chronic asthma in children show that most pediatricians were having similar pattern in their clinical practice. The participants were different with regard to the test of choice in asthmatic children by responding “complete spirometry” (52.7%) and “peak expiratory flow only” (47.3%). The distribution of responses to 4 statements (1, 3, 4 and 7) showed statistically significant difference (*P* < 0.001), whereas the responses to 3 statements (2, 5 and 6) were not significantly different (*P* > 0.05).

### Association between position, practicing experience, number of patients visiting a day, and knowledge and practice of spirometry

The three positions (consultant, registrar and senior resident), the three categories of practicing experience (≤5 years, 6-10 years and >10 years) and the number of patients consulting per day (≤5, 6-10 and >10 patients) were not statistically significantly associated (*P* > 0.05) with the distribution of responses to all the 17 statements of both knowledge and practice of spirometry. This shows that knowledge and practice were independent with the three study variables: position, duration of practicing experience and number of patients visiting daily.

## Discussion

Spirometry is considered a simple, noninvasive screening test, but careful consideration should be paid to a number of aspects, including optimal test performance, adherence to standard acceptability, repeatability criteria, accurate and proper interpretation of the results. In spite of all these factors, the fact is that there is lack of literature to describe the knowledge and practice of spirometry among pediatricians. Therefore, the idea was to assess the knowledge and practice of spirometry among pediatricians in their clinical practice at various tertiary hospitals in Riyadh, Kingdom of Saudi Arabia.

The present study indicates that about 37.6% to 56% of study subjects have responded “very useful”, 38% to 52.5% responded “fairly helpful” and 2.8% to 9.9% responded “not helpful”, towards knowledge of spirometry for diagnosis, prognosis, grading severity, monitoring and patient compliance. Similarly, it was reported that spirometry measurements can be fundamental to medicine as are pulse, blood pressure, temperature, height and weight measurements, and therefore it could be considered in physical examination as vital sign.[12] In addition, conflicting indications for office spirometry have appeared in medical literature, from recommendation that spirometry be incorporated into the routine examination in clinical settings.[13]

Our results also indicated that the participating pediatricians were not having similar knowledge and confidence in interpreting spirometry results relating “while reviewing the flow volume loop/flow time curve” and shows a distribution response on 4-point scale [never (32.4%), rarely (19.6), sometimes (23.5%) and all the time (24.5%)]. This shows that not only the level of knowledge was incomplete but also the confidence was poor. In agreement with our results, Chan *et al.*[14] reported that the usefulness of information from flow volume (FV) loops should be put into context and the general knowledge about the use of spirometry among primary care physicians was poor, but improved after workshop training.[15] Similarly, we observed that 86.1% of our study subjects have responded negatively to the question, “Are you up-to-date with the current knowledge of spirometry in pediatrics”. The results of the present study shows that 50% of the pediatricians were not confident, 38.9% were fairly confident and only a small percentage (11%) of pediatricians were confident “about interpreting PFT reports”, which obviously restricted pediatricians from using PFT in their daily clinical practice. Our results correlate with the results observed by Kimnesky *et al*.[15]

Moreover, while asking about how frequently they were suggesting spirometry among patients suffering from acute or chronic respiratory disease, 55.7% of pediatricians responded that spirometry is not necessary while 44.3% of them replied positively and recommended the test. However, it was observed that about 85% of clinicians suggested spirometry for the diagnosis and follow-up of acute/chronic respiratory diseases.[16] The most probable cause of suggesting spirometry was the level of education both in clinicians and patients.

It has also been reported that GPs utilized spirometry in daily practice not only for the diagnosis of respiratory diseases but also for management purpose. Similarly, our results also indicate that pediatricians used spirometry for the management purpose as reported by Eaton *et al.*[17] and Jones *et al*.[18] In addition, O'Dowd *et al.*[19] determined physician-related and practice-related factors that were associated with the use of spirometry in the evaluation of new asthma patients. General practitioners believed that such testing provided the data necessary for diagnosis. Similarly, in our study, we observed that all pediatricians responded that spirometry is an essential tool for the diagnosis of respiratory problems in children.

## Strength and Limitation of this Study

This was the first study that assessed the knowledge and practice of spirometry among the pediatricians of Riyadh, Kingdom of Saudi Arabia. The results of this study provide an opportunity to recommend appropriate periodical continuing medical education programmes among pediatricians so as to update their knowledge in spirometry. However, a limitation of this study was that it has used a subjective instrument with moderate sample size to assess the outcomes.

## Conclusion

The study results indicate that pediatricians were lacking the knowledge and practice of spirometry in their clinical practice. Hence, it was suggested that pediatricians should attend periodical training, workshops and continuing medical education programmes to enhance their knowledge, especially during their pediatric residency training programmes, as spirometry is one of the essential tools in clinical practice.
